# The benefits of using Sentinel WebDashboard in medicine: IT solution for monitoring and treatment of patient with liver cirrhosis

**Published:** 2014-06-25

**Authors:** SR Dumitrescu, D Popescu, VL Purcarea, LC Albu

**Affiliations:** * Faculty of Automatic Control and Computers, Polytechnic University in Bucharest; ** Carol Davila" University of Medicine and Pharmacy, Bucharest; *** University Emergency Hospital, Bucharest

**Keywords:** liver cirrhosis, upper gastrointestinal bleeding, Service Oriented Architecture, Sentinel WebDashboard

## Abstract

Abstract

The global assessment of the evolution of a disease in a certain geographical area or a specific domain is useful in the medical research for the preparation of practice guidelines/protocols used in the hospitals. Cirrhosis is one of the most common disorders seen today, occupying a significant place in the gastrointestinal pathology. The disease is the final stage of various affections in terms of etiology and morphology. The most frequent subjects treated on this topic are those related to the etiopathology and early diagnosis. Given the current interest in this matter and considering that UGS (upper gastrointestinal bleeding) in liver cirrhosis is a common complication and potentially fatal, the medical research found some very useful conducting retrospective studies in this area. The purpose of our study was to create an IT system implemented with Sentinel WebDashboard, which could increase the medical performances in diagnosis, monitoring and treatment of a disease. We tested our solution on a medical data set containing information about the patients with liver cirrhosis. The solution facilitates the access of the physicians to the databases containing complete information about the patients, offers the possibility to monitor the evaluation of their health and also aids physicians in optimizing the medical procedures and improve the diagnostic methods. It also offers the advantages of a web application: it does not require the installation on the client side, being accessible anytime, anywhere via a web browser, laptop, Smartphone or tablet.

Abbreviations UGS = upper gastrointestinal bleeding; UGE = upper gastrointestinal endoscopy; SOA = Service Oriented Architecture

## Introduction

The medical information collected in hospitals can be used to create hospital morbidity reports, to assess the availability and adequacy of services provided (e.g. can generate reports on the number and type of patients who can develop disease risk factors, etc.), can analyze if the medical errors exist in the treatment dealing and also in the comparison of the data with information provided in the previous guidelines. Considering all these aspects, the paper proposes an IT solution to help the doctors and the medical researchers, by providing a system which is accessible in a real time (without requiring the user’s involvement in information management database), that can securely integrate all the medical information and can help them in the diagnosis, treatment and monitoring of a disease.

 The first phase of the research was to analyze the objectives and to establish the implementation technologies. The next phase consisted in the medical documentation, which was extremely useful both for understanding the principles underlying the case study but also in medical data (used in testing the solution) analysis.

 About Sentinel WebDahboard

 Sentinel WebDashboard is a module integrated in Sentinel application, a software offered by Axway Company. Sentinel enables the user to discover, monitor and analyze the data as it flows across his entire enterprise ecosystem. Sentinel monitors both Axway and third party applications and systems, giving the client a clear visibility into all the data flows throughout his extended value chain [**1**].

 One of the most powerful features offered by Sentinel is the possibility of building intuitive dashboards that enable the user to monitor and capture global events from both Axway and third party applications and systems in real time.

 Axway Sentinel is based on a service-orientated architecture (SOA) and uses the HTML5 technology. It is a scalable, versatile and collaborative solution. 

 The application provides the advantage of developing reports and dashboards from multiple and heterogeneous data sources. The information can be accessed anytime, anywhere, 24 hours per day. The data filtering and selection is quick and easy. The HTML 5 self-service interface supports custom views and it can be acceded in real time by using a web browser or a mobile device. 

 The data access is secured by defining privileges/rights to the users:

 • Manage Web dashboards, access, reports and database – allows the user to control user-related data, configure database connections and set up reports and dashboards

 • Create Web dashboards and reports – allows the user to specify the data dictionaries, build reports and dashboards

 • View Web dashboards and reports – allows the user to view defined reports and dashboards, to customize and apply filters

 Summarizing, the Web Dashboard offers benefits such as flexibility, interoperability, agility, collaboration, usability and security.

 1. Service oriented architecture

 A service-oriented architecture represents a new model for the creation of evolving distributed applications. Services are distributed components that provide well-defined interfaces that process and deliver XML messages.

 According to [**2**] the SOA definition is "The policies, practices, frameworks that enable the application functionality to be provided and consumed as sets of services published at a granularity relevant to the service consumer. Services can be invoked, published and discovered, and are abstracted away from the implementation by using a single, standards-based form of Interface." 

 Other earlier definitions state that the "Applications must be developed as independent sets of interacting services offering well-defined interfaces to their potential users. Similarly, supporting technology must be available to allow the application developers to browse collections of services, select those of interest, and assemble them to create the desired functionality" [**3**]. In [**4**], a service is described as "A course-grained, discoverable software entity that exists as a single instance and interacts with applications and other services through a loosely coupled (often asynchronous), message-based communication model".

 Among the advantages that SOA could bring, the following can be considered:

 • Basic functions are concentrated around a service that runs independently of the application, once it is developed and out of the testing phase and debug, it represents a resource available to any project; this way it decreases the implementation time;

 • Any changes that lead to an improved workflow within a service are reflected immediately in all the applications that use it; the development time decreases;

 • Facilitates collaboration and information sharing throughout the organization and with external partners;

 • Keeps the complexity of business-to-business integration, significantly reducing costs and raising the level of business technology.

 Taking into account all the advantages offered by SOA, we consider that the implementation of such architecture would help the integration of various medical systems and services in a secure manner without endangering patients and providing confidential data.

 2. Implementing a solution for treatment in Sentinel WebDahboard and monitoring the patient with liver cirrhosis

 Considering all the technological advantages offered by the Sentinel WebDashboard, we believe that it outcomes all the needed features for the implementation of an IT system that can increase the medical performances in diagnosis, monitoring and treatment of a disease.

 Firstly, we have built a database that meets all the necessary conditions for the implementation and testing the solution with Sentinel WebDashboard.

 The medical data set was collected in a retrospective observational study in a group of patients with cirrhosis hospitalized between January 1 and December 31, 2010.

 The study includes a total of 298 patients who were admitted to the clinic during this period. The aim of the study was a comparative evaluation of clinically and laboratory results for cirrhotic patients with and without UGB.

 Based on this assessment one can highlight the risk factors for UGB, the evolutionary particularities depending on the stage of the disease, source and severity of bleeding, the presence of associated diseases disfavored for upper UGB, the pathogenic mechanisms involved in the onset of the first episode of UGB and re-bleeding, the treatment type and timing of its establishment [**5-7**].

 The processed data were obtained by consulting computer archives of internal medicine clinic, observation sheets and the results of laboratory investigations.

 Initially, the medical information was gathered in an xls file. After analyzing this file and the relationships between all the variables used (patient data, clinical analysis, treatment, medication, etc.) we have created a relational database schema for the implementation of the database. 

 Based on the analysis of the data set from the retrospective study, we tried to highlight in reports and dashboards the following information:

 • distribution of upper gastrointestinal bleeding in cirrhosis according to sex, age;

 • highlighting the correlation between different etiologies of liver cirrhosis and the presence of variceal gastrointestinal bleeding;

 • prognostic classification (according to Child-Pugh score) patients;

 • determine the number of upper gastrointestinal bleeding and the age range where they are most common;

 • patients distribution according to the size of portal vein and splenic vein obtained from abdominal ultrasound;

 • patients distribution according to the size of spleen obtained from abdominal ultrasound and patients distribution according to the amount of ascites visualized on the abdominal ultrasound; .

 • table with the list of investigations and diagnostic procedures for cirrhosis , highlighting changes in biochemical parameters, such as platelets, hemoglobin, SGOT, SGPT

 • table with ultrasound and endoscopic investigations list, highlighting the values for ultrasound portal vein (PV), ultrasound splenic vein (SV), and those for upper gastrointestinal endoscopy (UGE);

 • table with information regarding the treatment performed during a hospitalization, presenting a regimen followed for each patient: medication treatment, Blackmore probe treatment, UGB treatment;

 • prognosis and hospital mortality.

 Implementing the solution

 The first step was to create the schema of the database and to choose its type. Sentinel WebDashboard supports more than 200 database versions. The study was conducted on a MySQL 5 database server, which was already integrated in the software package provided by Axway [**1**]. 

 Once the database was created, we defined a world that was a collection of data necessary for the connection to the database. The parameters that defined a world were the driver, the URL for the database and the credentials for logging (consisting of username and password).

 The next action was to create a data dictionary that represented a selection of data from the database on which the reports, and ultimately the dashboards will be based.

 Having the data dictionary, one could create a report. This is a representation of the data set in a variety of forms. There are many options for a report implementation that can ensure the creation of complex graphical reports, based on data extracted from multiple tables.

 After entering the required data to create a new report (name, description, data dictionary), we had the possibility to choose the fields that will be used to create the report, but also the option to create new fields.

 Calculated fields were derived from multiple other fields and could be used on the dashboard as such to simplify the sql statement (they were similar to prepared sql statement). Most of the reports in our implementation were built by using calculated fields.

 The final step was to create the dashboard, which was a document that displayed the data extracted by defining sets of data used in the report.

 The dashboards are easy to use, built to follow the logical steps of tracking patient throughout his hospitalization, but in the same time, also provide statistical information about the disease. 

 The four dashboards are connected by dynamic links giving the user the possibility to navigate from one dashboard to another, so that the access to the information is done in a logical way.

 Basically, the application offers the advantage of building a medical history of each patient (recorded in the database) and his consultation whenever needed.

 Users can maximize each report by clicking the box in the upper right corner, they can print a report in pdf format or can even export it in an xls format file (for tables). Also, for each table, the physician can search a specific patient (to view only his medical analysis) by applying a filter on column IdPatient.

 In order to better understand the dashboards’ functionality and the way these can increase the medical performance, a short description of each will be made.

**Fig. 1:**
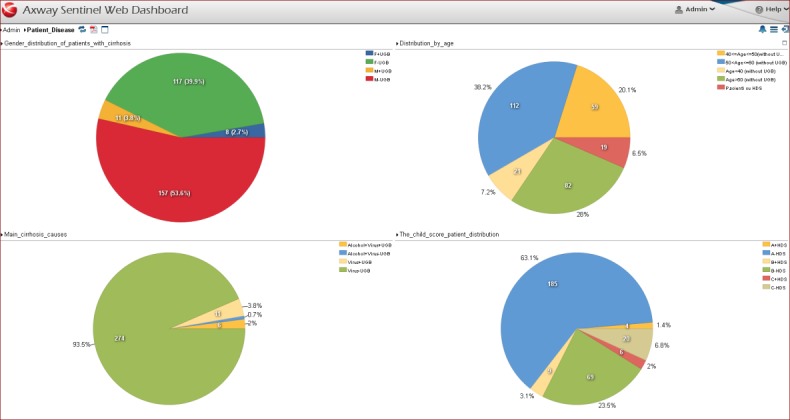
Dashboard 1

**Fig. 2:**
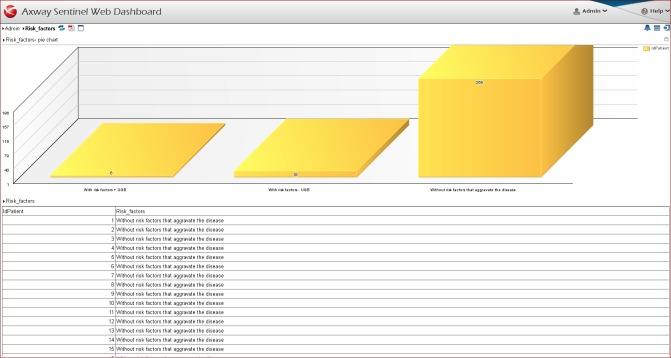
Dashboard 2

 Dashboard 1 - Patient disease (**Fig. 1**) consists of four reports showing statistical information about the distribution of cirrhotic patients with or without UGB depending on many factors, such as [**5,7,8**].: 

 - Gender distribution of patients with cirrhosis -> based of the first report one can differentiate four groups of patients (men with or without UGB and women with or without UGB) and one can determine in which of this causes the rate of the disease, the evolution is higher;

 - Distribution according to age -> based of the second report, one may establish the age groups for the two groups of patients (men, women) that can be considered as additional risk factors for the occurrence of upper gastrointestinal bleeding;

 - Main cirrhosis causes -> the third report highlights the distribution of patients according to etiology (viral, alcohol or combined); 

 - The child score patient distribution -> the fourth Report offers statistics on the repartition of patients according to the Child class to which they belong, which is basically a classification of the severity of the disease;

 Dashboard 2 - Risk Factors (**Fig. 2**) consists of two reports: a table and a pie chart. The table contains information regarding the risk factors that could aggravate the disease for each patient [**5,8**]. The risk factors are considered an important element in monitoring the evolution of the disease, because they cause a high rate of patient decompensation.

**Fig. 3:**
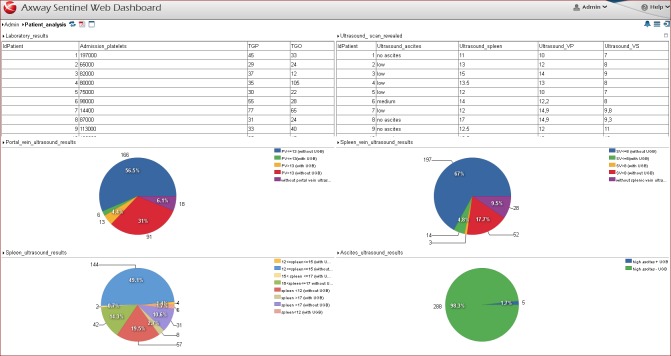
Dashboard 3

 Dashboard 3 – Patient analysis (**Fig. 3**) provides the most important analysis (clinical and laboratory). This data offer important information about the patient's condition at the time of his admission [9-12].

**Fig. 4:**
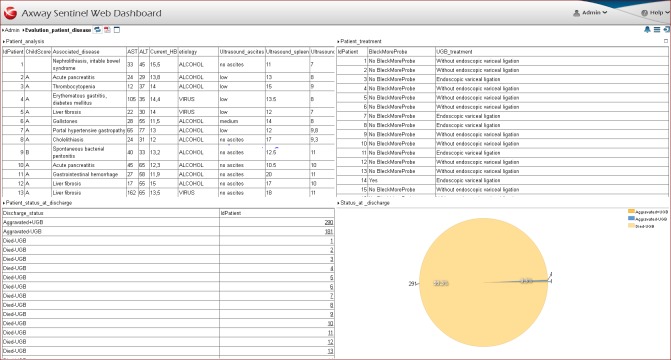
Dashboard 4

 Dashboard 4 – Evolution patient-disease (**Fig. 4**) provides a global vision about the therapy management applied to each patient [**7,13,14**]. Based on the report Patient status at discharge, the user has the possibility to assess the patient’s state of health during hospitalization. In addition, the pie chart Status at discharge evaluates the prognosis of patients and hospital mortality rate.

## Conclusions

This solution enables a secure integration of all medical information related to diagnose, treatment and monitoring of the disease. 

 The tests were done on patients with liver cirrhosis, but the implementation can be extended to any kind of disease. 

 The use of this application facilitates the identification of patterns for medical history evolution. It can be considered a very useful tool for the establishment of protocols and therapeutic guidelines.

 The implementation of this solution can improve the performance in healthcare services, but it can also be considered a good support for research and education.
